# High-gamma mirror activity patterns in the human brain during reach-to-grasp movement observation, retention, and execution—An MEG study

**DOI:** 10.1371/journal.pone.0260304

**Published:** 2021-12-02

**Authors:** Alexander M. Dreyer, Jochem W. Rieger

**Affiliations:** Department of Psychology, Carl von Ossietzky University Oldenburg, Oldenburg, Germany; Justus Liebig Universitat Giessen, GERMANY

## Abstract

While the existence of a human mirror neuron system is evident, the involved brain areas and their exact functional roles remain under scientific debate. A number of functionally different mirror neuron types, neurons that selectively respond to specific grasp phases and types for example, have been reported with single cell recordings in monkeys. In humans, spatially limited, intracranially recorded electrophysiological signals in the high-gamma (HG) range have been used to investigate the human mirror system, as they are associated with spiking activity in single neurons. Our goal here is to complement previous intracranial HG studies by using magnetoencephalography to record HG activity simultaneously from the whole head. Participants performed a natural reach-to-grasp movement observation and delayed imitation task with different everyday objects and grasp types. This allowed us to characterize the spatial organization of cortical areas that show HG-activation modulation during movement observation (mirroring), retention (mnemonic mirroring), and execution (motor control). Our results show mirroring related HG modulation patterns over bilateral occipito-parietal as well as sensorimotor areas. In addition, we found mnemonic mirroring related HG modulation over contra-lateral fronto-temporal areas. These results provide a foundation for further human mirror system research as well as possible target areas for brain-computer interface and neurorehabilitation approaches.

## Introduction

Ever since the first descriptions about mirror neurons in monkeys were reported [1], the existence of a human mirror neuron system and especially its functional role has been vividly debated [2]. In short, mirror neurons are neurons that show similar responses to action observation and action execution, especially for meaningful, goal-directed actions. These neuronal response patterns have been suggested to represent automatic direct mapping of the observed movement to the observers motor repertoire helping the observer to understand the action intention of other individuals [3]. As single neuron studies in humans are scarce, only few reported actual mirror neuron findings as of yet (for example: [4]). Hence, human mirror neuron studies tend to show a distributed mirror neuron system and report results on a larger spatial scale, either investigating mirror neuron sites from functional magnetic resonance imaging (fMRI) or as electrophysiological signals in sensors which show similar response properties as classic mirror neurons during action observation and execution. For example mu power decreases in central electroencephalogram (EEG) electrodes [5], beta power attenuation in magnetoencephalogram (MEG) sensors over sensorimotor cortex [6], or high-gamma (HG) power increases in electrocorticography (ECoG) where electrodes are placed directly on the cortex [7, 8].

fMRI studies investigating the neural circuits involved in hand movement observation and imitation characterized inferior parietal lobule, inferior frontal gyrus, and premotor cortex as mirror neuron sites involved in all phases of movement observation, imitation preparation and actual imitative execution [9, 10]. These mirror neuron sites coincide with ventral and dorsal sensorimotor areas thought to be involved in reach-and-grasp control [11–16]. Ventral-premotor and anterior parietal cortex involvement was reported for finger movement imitation [17] in fMRI. While the existence of a human mirror neuron system became evident [18], investigations into involved cortical areas and their functional properties continue.

Perry et al. [7] investigated properties of the human mirror neuron system by analyzing HG responses using ECoG. Patients watched videos of different object-grasps, remembered the observed object and grasp type during a short delay period, and then executed the memorized grasp. Based on their results, Perry et al. [7] defined *pure mirror sites* with significant high gamma activation only during observation and execution of a movement. HG activity in ECoG is correlated with neuronal spiking activity [19], and as such is thought to reflect a signal similar to the single neuron spike activity used to investigate mirror neuron activity. ECoG (and MEG) HG activity, however, arises from population activity. This creates an ambiguity in its interpretation. Theoretically, HG activity could reflect spike activity in different neuronal populations that are in close proximity to the same electrode during movement observation and execution. Conversely, recordings from single mirror neurons, via single-cell spike recordings, could unambiguously attribute features of its spike activity to a particular neuron. Following the definition of Perry et al. [7], we will denote sensors that show overlapping significant HG modulation during movement observation and execution at a population level as mirror sensors, while keeping the above mentioned caveats in mind. In addition to *pure mirror sites*, Perry et al. [7] defined *mnemonic mirror sites* that showed elevated activation during observation, retention, and execution. Both, pure and mnemonic mirror sites were reported in motor cortex, somatosensory cortex, parietal regions and in the inferior frontal gyrus.

While Perry et al. [7] report only increases in HG activity during action observation and execution, single-neuron studies draw a more complex picture. Mukamel et al. [4] found cells responding to action observation and execution in medial frontal and medial temporal lobe. A subgroup of these cells responded with an increased firing rate during action execution and a decreased firing rate during action observation. Mukamel et al. [4] suggested that such opposite patterns of activity might serve a control function to inhibit unwanted imitation or to maintain self-other differentiation. Similar observations using fMRI were made by Gazzola et al. [20] who found voxels with augmented BOLD activity during action execution and significant BOLD reductions during action observation in primary motor cortex and suggested this suppression to originate in supplementary motor area (SMA) and middle cingulated cortex as a decoupling function during action observation. Studies have also found suppression in several cortical areas during preparation of observing an action with the intent of imitating it [21].

While the modulation of HG and spike activity by action observation is a defining feature of the mirror neuron system, the direction of the effect (reduction or increase) and its functional implication is less clear. In the current study, we used a slightly adapted version of the paradigm run by Perry et al. [7]. In addition to analyzing the spatial pattern of HG responses over the whole cortex, our goal was to replicate the mnemonic mirror activity introduced by Perry et al. [7] and to test whether such responses can be found in other areas that were not available in the spatially limited ECoG recordings. We used the whole-head coverage of MEG recordings to gain additional insights into the spatial activity patterns during observation, retention, and execution of natural human reach-to-grasp movements. Our main focus is activity in the HG band which has been shown to reveal task-related neuronal population activity in ECoG recordings at a good spatio-temporal resolution [22] and to be correlated with neuronal spiking activity [23, 24].

While MEG does have a high dynamic range which in principle allows analysis of high frequency signals, MEG studies on HG activity are still scarce, as the signal-to-noise ratio of HG signals is rather low in MEG, thus requiring a high number of repetitions and noise-free recordings. There are a number of MEG studies showing HG responses to finger movements [25], foot movements [26], or reach movements [27], but we are not aware of any study directly comparing movement observation and execution responses in the HG range.

An additional extension to previous studies is that we used natural human grasping movements which should provide results with a high ecological validity. However, such natural, less controlled movements can also increase noise, especially in MEG recordings [28]. We employed a custom made MEG and MRI compatible motion-tracking system to have better control over wrongly executed movements and to be able to analyze the MEG data in close relation to actual instead of instructed behavior.

To the best of our knowledge, this is the first study explicitly characterizing HG mirror-like responses to natural reach-to-grasp movements with MEG. Our results for movement observation and execution show a combination of HG power increases and decreases in sensors over different cortical locations including ventral and dorsal sensorimotor and occipito-parietal areas. Sensors over several cortical areas show mirror properties in the HG range. Mirror-like activity in central sensorimotor areas was limited to a short interval before the grasp. We found consistent HG activity decreases in sensors over inferior frontal and superior temporal gyrus during movement observation, retention and execution, potentially hinting at mnemonic properties in these areas. Our results reveal the complex spatial patterns of overlapping HG modulations during movement observation and delayed imitation—putative mirror system responses.

## Material and methods

### Participants

The study was approved by the local ethics committee at the University of Oldenburg (Approval Numer: 2018/072). Written informed consent was obtained from all participants. We recorded 15 healthy volunteers (7 female) with a mean age of 26 years (range: 21–35). All participants were right-handed according to the Edinburgh Handedness Inventory [29].

### Experimental design

The experiment is a slightly adapted version of the paradigm used by Perry et al. [7]. Participants were comfortably seated in the MEG chair with three everyday objects (cup, bottle and pencil) sitting on a table in front of them. Between trials and during non-movement periods, participants were instructed to place their hands on their lap and avoid movement.

**Fig 1:**
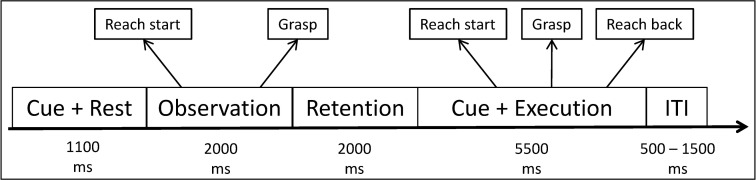
Experimental paradigm: Each trial starts with an auditory cue, followed by a short baseline interval. Subjects then watched a video of a reach-and-grasp movement, remembered the movement / object and executed the same reach-and-grasp movement after another auditory cue.

The trial structure is depicted in Fig 1. Each trial started with a short auditory cue followed by a background image on the screen which constituted the baseline period of 1100 ms. Then a short video clip of a person grasping one of the three objects with their right arm using an object-specific grasp (cup: handle grasp; bottle: whole-hand grasp; pencil: pinch grasp) was shown. The participants had to observe the video and remember the object and grasp type. For the rest of the trial, the background image was shown again. 2000 ms after the video, another auditory cue was presented signaling the participants to reach for and grasp the object seen in the video with their right hand, hold the grasp for a short period of time without lifting the object, and move back to the rest position. For this self-paced movement, participants were allowed to take up to 5500 ms. The inter-trial-intervals were randomly set between 500 ms and 1500 ms. All stimuli were presented using Presentation (Neurobehavioral Systems, Berkeley, CA, USA).

This trial structure led to three main periods of interest. The observation period during which participants passively observed a reach-to-grasp movement, the retention period during which participants remembered the object and the grasp type they have seen, and the execution period during which participants executed a reach-and-grasp movement towards the remembered object using the remembered grasp type. Note that the subjects were instructed to simply grasp the object without lifting or moving it, similar to the movements visible in the videos.

Overall, a recording session took about 1 hour, in which we recorded 3 blocks of around 20 minutes length with 111 trials each. In total each participant performed 333 trials. The video order was pseudo-randomized (37 repetitions per each of the three objects per block) and object positions were changed during the blocks so that each object was in each position for one block. Before the actual experiment, we ran a few training trials until the subjects felt comfortable with the task.

### MEG recordings

We recorded MEG data using a 306-channel whole-head MEG system (Elekta Neuromag Triux, Elekta Oy, Helsinki, Finland) located inside a magnetically shielded chamber (SK3B with an additional layer, Vacuumschmelze, Hanau, Germany). MEG signals were recorded without internal activate shielding, at a sampling rate of 1 kHz, and online filtering between 0.03 Hz and 330 Hz. The dewar position was set to 68°. Continuous head position tracking was applied using 5 head position indicator coils that were attached to the participants’ head. Coil positions and anatomical landmarks (nasion, left and right pre-auricular points) were digitized using a Polhemus Fastrak (Polhemus, Colchester, VT, USA).

In addition, we recorded electromyogram close to the subjects right wrist, vertical and horizontal electrooculogram (EOG), and electrocardiogram (ECG).

Experiment timing was verified and adjusted by splitting and feeding the sound signal of our auditory cues directly into the MEG amplifier as well as the voltage-signal from a photodiode attached to the projection screen to monitor video on- /offsets.

### Movement tracking

Correct execution of the specific grasp movements was monitored and verified via video recordings of arms and hands during the whole experiment. In addition, we placed an accelerometer on the right index finger and a custom made system of gyroscopes along the right arm of the participants. This MEG and MRI-compatible arm tracking system was developed by Shirinbayan et al. [30] and successfully used for arm movement reconstruction [31].

Only correct trials, in which subjects grasped the correct object with the correct grasp type and neither grasped before the grasp cue nor exceeded the trial time, were used for further analysis.

### MRI acquisition

Following the MEG recordings, we obtained structural MRI images from all but one participant, who was feeling unwell in the MRI scanner. We used a Siemens Magnetom Prisma 3 T whole-body MRI scanner (Siemens, Erlangen, Germany) with a T1-weighted 3-D sequence (MPRAGE, TR  =  2000 ms, TE  =  2.07 ms) with a slice thickness of 0.75 mm. Individual brain surfaces were reconstructed from the T1 images using freesurfer [32].

### MEG analysis

As a first step, we preprocessed all raw files by using the manufacturer supplied Maxfilter software and applying temporal signal space separation to attenuate magnetic interference not originating from brain sources, continuous head movement correction, and data transformation to a default coordinate system with equal origin for all subjects. The quality of the head movement correction was very high as the goodness of fit of head position indicator coils was over 0.99 (on a scale from 0 to 1) for all subjects at all times. The average sample to sample (1 Hz sampling rate) 3D head movement distance calculated from the indicator coils was 0.17 mm (SD = 0.24 mm) with 99.8% of the distances being under 2 mm.

For further analysis we used MNE-Python (version 0.20). We applied a band-pass filter between 0.1 Hz and 250 Hz and down sampled the MEG data to 500 Hz. Then, we visually inspected the frequency spectra of the continuous data and found several noise peaks in individual frequencies which we suppressed using zero-phase, finite impulse response notch filters with half-amplitude cutoffs at +/- 0.25 Hz to suppress power line noise and its harmonics (50, 100, 150 Hz), the noise produced by our camera (60, 120, 180 Hz), and two additional noise peaks with unidentified sources at 48 Hz and 62.5 Hz. In addition, we corrected potential heartbeat and eye movement artifacts using independent component analysis (‘fastica’) as implemented in MNE-Python on the filtered data. We used recorded EOG and ECG signals to identify the independent components reflecting the corresponding artifacts.

### HG extraction

We picked HG boundaries (65–95 Hz) in a range that has been shown to produce task-related effects in prior motor-related MEG studies [26, 33–35]. While higher upper boundaries for the HG band have been established in ECoG recordings (also in [7]), HG activity in frequencies beyond 100 Hz is hardly detectable with MEG. However, because amplitude variations of spike-correlated HG activity can be observed over a broad frequency band [24, 36], the narrower smaller HG band observable in MEG should reflect effects in the wider HG band that can be recorded in ECoG. We further down sampled the MEG data to 200 Hz for HG power extraction to reduce computational resource demand.

We focused our analysis on the planar gradiometers as the noise level of the magnetometers was too high for HG analysis. We band-pass filtered the sensor time series in 10 Hz bands between 65 Hz and 95 Hz bands respectively and calculated the absolute value of the Hilbert transform of the band pass filtered signals. We combined planar gradiometers using the root-mean-square. Finally, we epoched Hilbert transformed signals around the video onsets, baseline corrected them with the mean of the interval -0.6 s to -0.1 s before video onset (comparable to Perry et al. [7]), and averaged over bands to obtain the broadband HG envelope signal.

### Epoching and statistical testing

We defined three epochs for each trial: observation, retention, and execution of the grasps. We derived the respective onset markers from the onset and offset of the video, and the onset of the movements as indicated by the gyroscopes. Note that the duration of the movement execution in the videos was constant over repetitions of the same grasp type but differed somewhat over the three grasp types. Moreover, the self-paced nature of grasp execution required single trial level definition of the execution marker timings. Therefore, we used the average absolute data from all gyroscopes placed along the arm to determine onsets and offsets of arm movements. In addition to the reach onset event, we extracted a marker for the grasp event, when the fingers were closed around the object without additional arm movements for observation and execution epochs. All markers served to cut epochs for the analysis of HG variation around the respective grasp execution event. For the analysis of HG variation over whole reach-and-grasp movement in observation and execution epochs we used 2 s windows. In addition, we cut 500 ms intervals before (pre-grasp) and after (post-grasp) the time point of the grasp closure.

In order to test for modulations of the baseline corrected HG activity, we calculated series of one-sample t-tests against 0 for each sample in the HG time series separately for each channel. Significant activation was defined as at least 100 ms of consecutive p-values with p < 0.05 similar to the significance tests used by Perry et al. [7]. As an alternative approach, we used the Benjamini-Hochberg false discovery rate correction procedure [37] to control the proportion of type I error in multiple comparisons. This approach resulted overall in very similar sensor patterns of significant HG responses and was even slightly less conservative. Therefore, we report only the results from the minimum number of consecutive samples test employed by Perry et al. [7]. In an approach to control false discoveries we interpret only significant sensor patterns that comprise at least three neighboring sensors.

### Sensor sensitivity maps

Individual subject brain surface reconstructions were coregistered with the MEG space. Then, volume source spaces and forward models were computed using MNE-Python [38]. The planar gradiometers we focused our analysis on have the advantage that the signal generating brain source they are most sensitive to is most likely directly under the sensor [39]. However, according to the Biot-Savart law the strength of the signal in planar gradiometer sensors depends on the distance and relative orientation to a given brain region. As a consequence, the region from which a gradiometer picks up signals has a spatial extent. In order to accommodate for this spatial uncertainty we augmented the simple inference (active brain region under gradiometer) by considering the physical forward model as a spatial prior for the spatial localization of the active brain area. Therefore, we calculated from the individual brain anatomies forward models which can be interpreted as sensitivity maps (MNE-Python function ‘sensitivity_map’) for individual MEG sensors. In order to account for the influence of individual variations in brain anatomy on the sensitivity maps we morphed the individual maps to a standard brain (freesurfer ‘fsaverage’ brain) for visualization. Note that the sensitivity maps are solely based on physical models which do not require model fitting. They have the advantage that they do not depend on free parameters that can be chosen by the user but lack the estimation of activation strength. Activation strength in source space is of no interest here, since we are only interested in the possible origin of effects we observed in sensor space.

## Results

### Movement data

**Fig 2:**
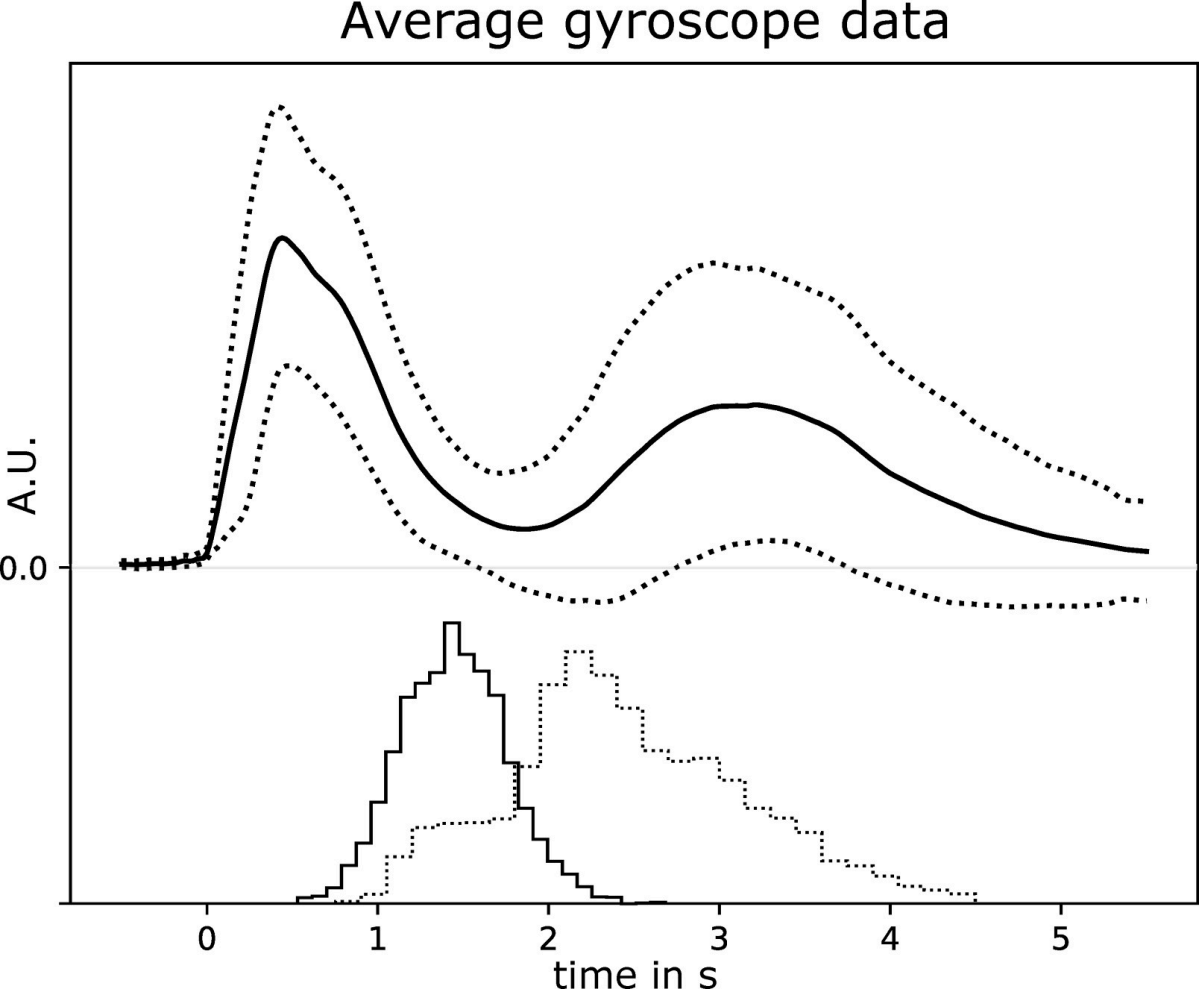
Average of absolute arm gyroscope signals (solid curve) +/- 1 SD (dotted curves) as an arm motion measure and histograms for grasp closure (solid line) and grasp-release (dotted line) markers. Data is aligned to the movement onset at 0 s. Note the considerable variability of the markers in the freely performed movements.

In 95.7% of the trials (4781 out of 4995) participants correctly performed the movements, i.e. correct object, correct grasp type, and correct timing. All following results are based on these correct trials. The average gyroscope time-course aligned to the movement onset and averaged over all arm gyroscopes, trials, and participants can be seen in Fig 2. In addition, histograms of the grasp closure and release times of all subjects are plotted at the bottom. The histograms show that there is considerable variability in the timings of the different movement phases. The average reaction time (time between auditory cue and movement start) was 272 ms (SD = 178 ms). Note that the reaction time is already realigned to 0 s in Fig 2. The average time between movement start and grasp closure was 1445 ms (SD = 311 ms), the average time the grasps were held was 1052 ms (SD = 690 ms), and the average time for the whole movement from start to rest was 4347 ms (SD = 761 ms). In the videos, the average reaction time was 247 ms and the average time between movement start and grasp closure was 1380 ms. These timings are similar to the movement execution, indicating that the participants executed the reach-and-grasp movements with a comparable dynamics as shown in the videos.

### MEG results

#### Time frequency analysis

**Fig 3:**
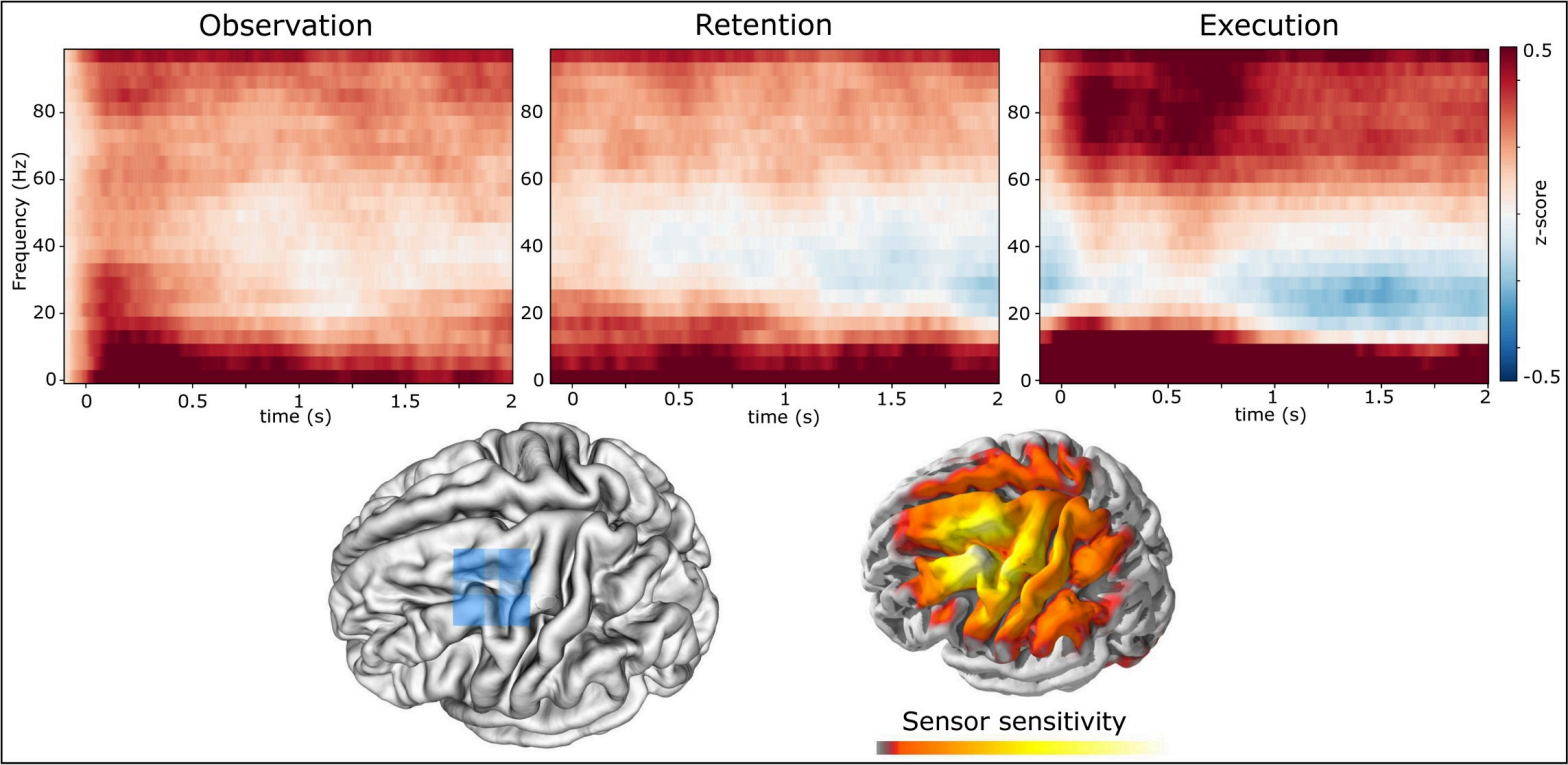
z-scored time-frequency response from a single gradiometer sensor located over left motor hand-knob. The three spectrograms depict activity in different frequencies during observation, retention and execution. Observed and executed movements were right hand movements. Observation and retention data is aligned to video on- / offset. Execution data is aligned to individual movement onsets. Bottom images depict the sensor location and the corresponding sensitivity map for this sensor on a standard brain.

Fig 3 depicts spectrograms of exemplary time-frequency responses for the three periods of interest in one gradiometer sensor located approximately above the hand-knob [40] of left sensorimotor cortex (contra-lateral to the movement). The spectrograms depict averages over all trials and subjects. The execution data is aligned to individual movement onsets. The power values are z-scored relative to the average baseline from 0.6 s to 0.1 s prior to the video onset.

On a descriptive level, the amplitudes in the alpha and lower frequency bands were increased during all three periods relative to the pre-video baseline. During the execution phase this increase in low frequency amplitudes likely reflects movement related evoked magnetic fields [41]. In addition, amplitudes in the beta band decreased during movement execution as expected [42]. Somewhat weaker band decreases can also be seen during the observation and retention periods which is in line with prior research [43, 44]. The stronger beta decrease at the end of the retention period could be related to movement preparation, as the movement period starts directly after the retention. HG power, between 65-95Hz, is increased during all three phases in this sensor. This increase is strongest during the execution phase, especially during the first second where the gyroscopes indicated strongest movement activity (see Fig 2). The brain images indicate the location of the sensor and its corresponding sensitivity profile calculated from the forward model. The sensitivity map can be interpreted as a probability map for the origin of the planar gradiometer signal in the brain. Note that the probability map is derived from a forward physical model of the planar gradiometer sensitivity and requires no inverse model fitting. In sum, this sensor over contra-lateral somatosensory cortex exhibited a spectral pattern that is well in line with previous reports, in particular with respect to the modulations in the beta and HG bands [45]. The subsequently reported results focus on the HG band activity acquired with the Hilbert transform and averaged over the band from 65–95 Hz, as described in the section 2.6.1.

#### High gamma around grasp closure

In order to obtain a more detailed picture of the HG activation modulations around the time of grasp closure in the execution phase, we used the movement tracking data to define the time of grasp closure in individual trials and aligned the HG time series to this marker for averaging single sensor data over trials. An analogous procedure was implemented to the single trial HG activity recorded during the observation phase. Therefore we extracted a marker for the time of the grasp closure from the videos.

**Fig 4:**
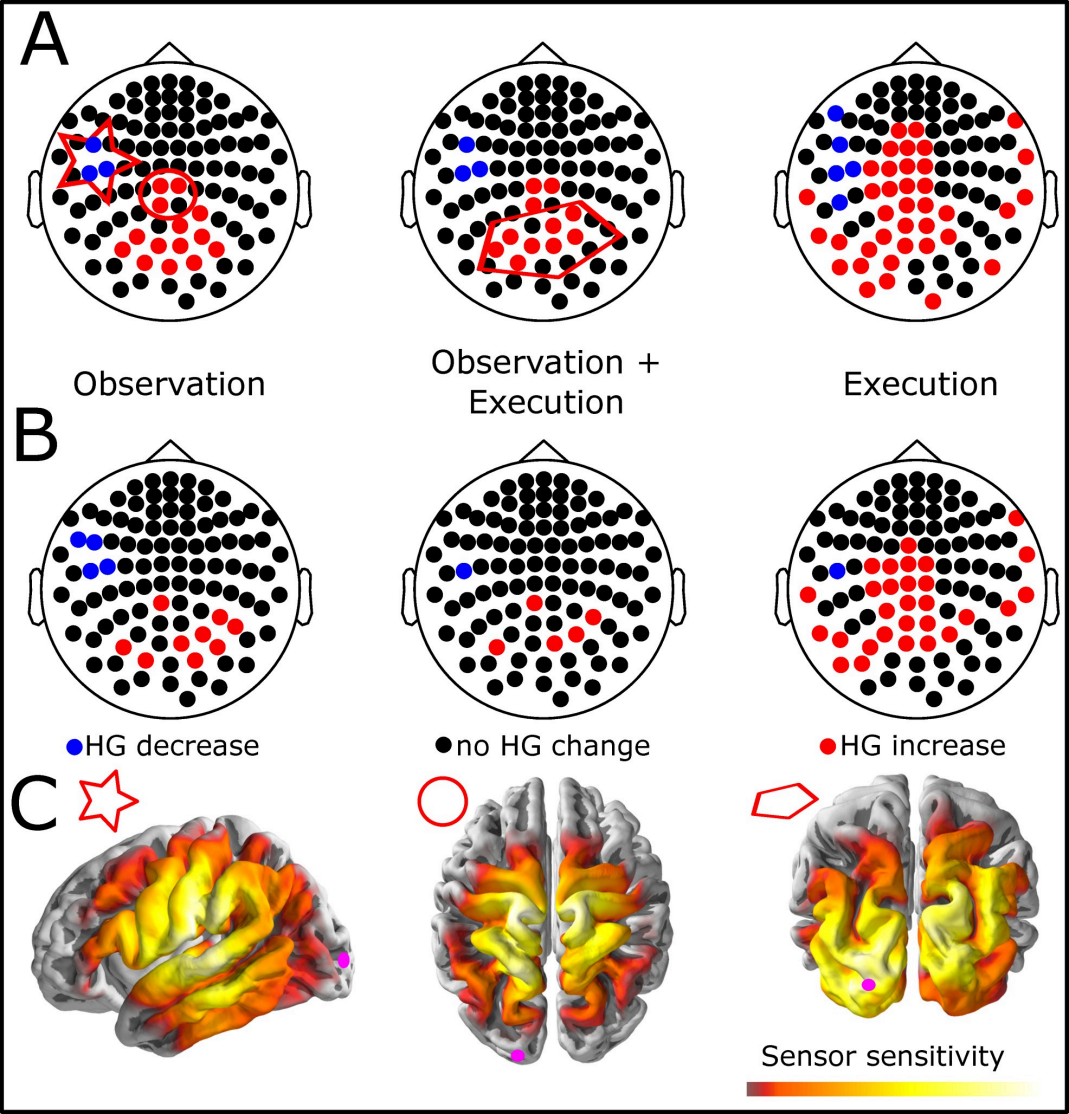
Overview of sensors with significant changes in high-gamma activity during Pre-Grasp (A) and Post-Grasp (B) observation and execution. Colored sensors denote significant changes (blue = decrease, red = increase) during at least 100 consecutive ms during the 500 ms before (A) and after (B) the grasp is closed around the object. Left panels show observation only, right panels show execution only, middle panels show the conjunction of sensors significant in observation and execution. (C) Sensitivity maps for the conjunctions of significant observation and execution sensors in A. The left map shows sensitivity distributions for sensors with HG decrease (marked by a star), the middle map for the three central sensors with HG increase (marked by a circle), and the right panels map for the eight occipito-parietal sensors with HG increase (marked by a pentagon). Sensor sensitivity distributions depict the brain areas from where the sensors likely picked up the HG signals. Brighter colors indicated higher sensitivity. The pink circle over left occipital cortex indicates 3D brain orientation.

We analyzed the 500 ms prior to the grasp closure (pre-grasp) during which grasp pre-shaping [46] and execution happen as well as the 500 ms after closing the grasp during which the grasp was held (post-grasp). Fig 4A shows the resulting patterns of significant sensors for the observation pre-grasp phase (left), execution pre-grasp phase (right), and the conjunction of significant sensors (middle). Relative to the pre-observation baseline, red sensors indicate a significant HG increase, blue sensors a significant decrease and black sensors no significant changes during the respective phases.

We found sensors with significantly increased HG activity in the *pre-grasp observation* phase in central midline sensors (Fig 4A left panel, red sensors in circle) with maximum sensitivity for signal from primary sensorimotor and superior parietal cortices (Fig 4C middle panel) and somewhat more posterior sensors (Fig 4A pentagon) with maximum sensitivity over parietal and occipital cortex (Fig 4C right panel). Moreover, we found HG decreases in contra-lateral mid-lateral sensors (Fig 4A blue sensors in star) with maximum sensitivity around the inferior sensorimotor cortices including the ventral premotor and higher order somatosensory cortices with some bias towards the latter (Fig 4C left panel).

During the *pre-grasp execution* phase we found additional HG increases over contra-lateral occipital and parietal cortices (Fig 4A right panel), potentially reflecting visuo-motor coordination of the grasp, and central with some contra-lateral lateralization around the dorsal primary and higher sensorimotor areas. Moreover, the HG power decrease in the contra-lateral left antero-central sensors now extends more anterior, potentially reflecting stronger involvement of ventral premotor cortices during the actual movement execution.

Following Perry et al. [7] we define the *pure mirror* related HG activity around the grasp closure as the conjunction of the significant sensors during the execution and observation phases which can be seen in the middle panel in Fig 4A. This conjunction includes the contra-lateral antero-central sensors with highest sensitivity over ventral sensorimotor cortices, higher order somatosensory cortices, and the parietal sensors. These brain areas are in concordance with previously reported nodes in a mirror neuron network in humans [7, 9] as well as in primates [47, 48].

During the *post-grasp phase* (Fig 4B) the HG activation increases are only found in subsets of the sensors that are active during the pre-grasp phase (Fig 4A). This is in line with the notion that placing a grip involves more visuo- and sensorimotor activation in primary sensorimotor and parietal areas than holding a grasp.

In the *post-grasp execution* phase (Fig 4B right panel) we find fewer contra-lateral sensors with a significant decrease compared to the pre-grasp execution phase (Fig 4A right panel). Only the most posterior sensor with highest sensitivity in higher order somatosensory areas in inferior parietal cortex exhibits the significant HG decrease in the *post-grasp execution* phase. This result is in line with the notion that activation decrease in this sensor cluster is related to the coordination of the grasp movement in higher order sensorimotor cortices. Interestingly, the extend of the significant decrease HG changes less in the observation phase (Fig 4A and 4B left panels). This suggests that observing an action may still be functionally different from performing a movement in these supposed mirror areas.

Accordingly, the conjunction of significant sensors in observation and execution phase reveals less sensors in the post-grasp compared to the pre-grasp phase (Fig 4A and 4B middle panel). However, this is mostly due to the reduced HG activation during the post-grasp execution, at least for the sensors with HG decrease over contra-lateral higher ventral sensorimotor cortices. Together, these results further support the notion of some functional differences between the overlapping motor execution and action observation related mirror activity.

In sum, focusing the analysis of the grasp interval of the reach-to-grasp task we found significant changes in HG activity in known mirror neuron areas [18], as well as coinciding areas known to be involved in reach-and-grasp movements [12]. However, we also found in our whole head MEG recording a previously unreported decrease of HG activation relative to a pre-observation baseline in sensors over ventral sensorimotor areas contra-lateral to the hand performing the grasp.

#### High gamma in reach-to-grasp observation, retention, and execution

**Fig 5:**
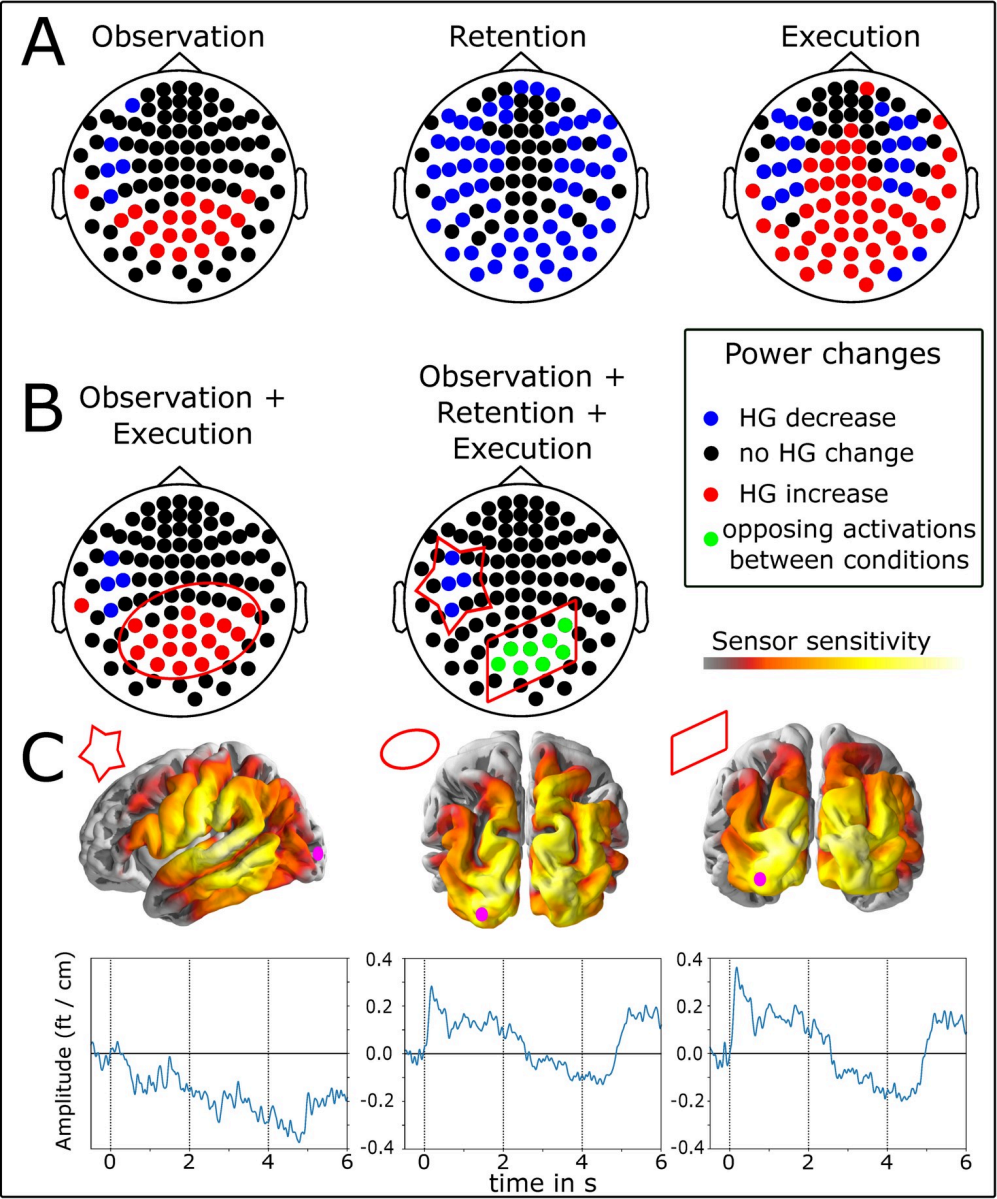
(A) Overview of sensors with significant changes in high-gamma activity during observation, retention and execution. Colored sensors denote significant changes (blue = decrease, red = increase) lasting for at least 100 ms in the 2 s windows of interest. (B) Conjunctions of the patterns shown in A. Blue and red indicate sensors with consistent effects between intervals (sensors with mirror properties). Green indicates sensors with opposite HG effects (decrease and increase) between the intervals. (C) Sensitivity maps for the three conjunction clusters shown in B. Brighter colors indicated higher sensitivity. The pink circle over left occipital cortex indicates 3D brain orientation. The left panel depicts sensitivity of the sensors with significant HG decrease (marked with star in B), the middle panel of the sensors with HG increase (marked by ellipse in B), and the right panel of sensors with opposing HG patterns (marked by parallelogram in B). The time courses show the corresponding average HG time course in each cluster. In the interval 0-2s participants observed the reach-to-grasp in the video, the retention interval lasted from 2-4s, and the interval from 4-6s seconds includes reach-to-grasp execution. The grasp closure occurred on average at 5.7 s in these plots.

Finally, we analyzed and compared the pattern of significant HG activations across all three tasks: reach-to-grasp observation, retention, and execution. We analyzed HG activation variations against the same pre-observation baseline and with the same statistical criterion that we used in the previous analyses. We included the complete 2 s of data from the observation and retention phase as well as the first 2 s of data from the execution phase aligned to the start of the movement. For the observation and execution phases this interval comprises the start of the reach and the grasp closure. In contrast to the previous analysis the trials are now aligned at the interval starts instead of the grasp closure. Fig 5A shows sensors with significant HG modulation for all three trial phases.

During *observation* of the reach-to-grasp interval significant HG activation increases and decreases follow a similar pattern as in the previous analysis focusing on the grasp interval only: a contra-lateral decrease of HG in mid-lateral to frontal sensors and an increase over more posterior sensors over parietal cortices (Fig 5A left panel). However, both HG decreases and increases exhibit a wider spatial extend than in the previous analysis where trials were aligned at the grasp closure. Additional parietal sensors with HG increases are sensitive to activation in bilateral posterior IPS which is known to play a role in reach movement control (Fig 5C middle panel; [12]). Additional contra-lateral sensors with HG decrease are most sensitive to HG activity in ventral premotor cortices known to be involved in the control of reach-and-grasp movements (Fig 5C left panel; [49, 50]) as well as higher order somatosensory cortices. We found no significant HG increase in the mid-central sensors over primary sensorimotor cortices, as opposed to the analysis in the previous section, which is likely due to the different temporal alignments (here reach movement start, previous grasp closure) and emphasizes the importance of proper alignment and movement tracking in self-paced movement experiments.

In the *execution* phase, the significant HG reduction in mid-lateral to frontal sensors is bilateral and extends over more sensors than during the observation phase (Fig 5A right panel). Bilateral activations during movement imitation tasks are in line with prior research [18]. The observation that the ipsi-lateral HG reduction was found when trials were aligned with the reach onset but not with the grasp closure suggests that ipsi-lateral reduction is related to the reach rather that the grasp. We found HG increases in most mid-central and posterior sensors which are located over primary sensorimotor and potentially dorsal premotor cortex with some contra-lateral lateralization. Moreover, a large group of sensors over parietal and visual areas showed significant HG increases. These findings are in concordance with the spatial distribution of a brain network for the control of visually guided reach-and-grasp movements [11, 16, 51, 52] as well as areas associated with action observation and imitation [18].

During the *retention* phase we found a prominent significant decrease of HG activity relative to the pre-observation baseline over posterior, bilateral lateral, and frontal sensors (Fig 5A middle panel) but no significant increases. This general reduction of HG activity could be a control mechanism that prevents the subjects from executing the movement before the start of the execution period, as suggested by Gazzola et al. [20]. Note that most of the central sensors over primary sensorimotor and premotor cortices, which showed an HG increase during execution, did not show any significant HG modulation during retention.

Sensors with putative mirror activity are shown in the left panel in Fig 5B. These sensor exhibited consistent significant HG increases or decreases during the observation and execution phases of the reach-to-grasp intervals. The sensor pattern is very similar to the pattern obtained in the peri-grasp interval (Fig 4A middle panel). The mirror activity comprises an HG increase in sensors over parietal cortex (marked by ellipse) and an HG decrease in mid-lateral sensors over higher order ventral sensorimotor cortices (marked by star). Average signal traces of these sensor clusters are shown in Fig 5C. The mid-lateral decrease can be seen throughout all phases with a slight rebound around the grasp closure (Fig 5C bottom left panel between 5–6 s) that remains below baseline. The activity pattern in occipito-parietal sensors with mirror-like activity is similar (Fig 5C bottom middle panel), but the HG activity during the observation and execution phases is significantly increased. We did not find HG activation increases over dorsal primary sensorimotor cortices. This suggests that the HG activation there is likely related to the grasp rather than the transportation phase and is therefore missing in this analysis.

The final conjunction we analyzed was between all three phases: observation, retention, and execution. Sensors with similar HG activation changes in all three phases were denoted *mnemonic mirror sites* by Perry et al. [7]. This conjunction reveals two sensor clusters (Fig 5B middle panel) that are similar to those obtained in the peri-grasp analysis: One cluster of mid-lateral sensors over ventral sensorimotor cortices with decreased HG activity relative to the pre-observation baseline. The direction of the HG effect is comparable in direction over all three phases but strongest during the retention and the beginning of the execution phase (Fig 5C). The posterior cluster of sensors over parietal cortex exhibits some ipsi-lateral lateralization but most notably, the HG activation is reduced during the retention phase and increased during the observation and execution phases. This suggests that overlapping brain areas support retention, observation and execution but the functions reflected in the HG-activity might change between memorizing and observation/execution.

## Discussion

The aim of our study was to characterize putative human mirror system related spatial patterns of HG activity accompanying realistic reach-to-grasp movement in whole head MEG measurements. Therefore we compared HG activity among three conditions: reach-to-grasp observation, retention of the grasp, and its execution. This allowed us to reveal mirror-like HG activity patterns and mnemonic mirror activity patterns as introduced by Perry et al. [7]. We found mirror-like HG activity in sensors over previously described mirror neuron sites and over brain areas known to be involved in the control of reach and grasp movements. Since broadband HG activity has been shown to be strongly correlated with single neuron firing [19] our population results hint at mirror neuron activity at these sites, but further single-cell studies are needed to unambiguously verify such claims. Our study also revealed decreases of HG activity in sensors over ventral sensorimotor areas relative to a pre-trial baseline. The decrease was particularly pronounced during the retention phase where it included additional sensors over parietal brain areas involved in reach and grasp planning. This parietal decrease may reflect neuronal activation reduction to prevent the execution of a memorized movement. To the best of our knowledge, such a distributed pattern of HG activity changes during movement observation, retention and execution has not been reported with MEG before.

In the intervals of reach-to-grasp movement observation and execution, we found a distributed pattern of mirror-like HG activation enhancements in sensors over parietal cortex along the IPS, the inferior parietal lobule, sensorimotor-cortex and more ventral, higher order somatomotor-cortices. This pattern is in concordance with previously described mirror neuron sites [47] and includes brain areas of the reach-and-grasp execution pathways [12, 53]. Moreover, aligning the HG timeseries to the grasp placement instead of the trial onset, revealed additional sensors with mirror-like HG modulations over central areas (see Fig 4A middle panel) in the interval just prior to the grasp. This HG activity likely originated from hand areas [40] in sensorimotor cortex suggesting mirror neuron activity in primary sensorimotor cortices, as well as in anterior intra-parietal areas which are known to play a role in grasp coding [12]. Note that both analysis variants were performed on overlapping data segments and differed only with respect to the temporal alignment of the recordings. This emphasizes the role of the alignment of the analysis with the actual behavior and the importance of recording the actual behavior during movement tasks to optimize the sensitivity of the analysis of neuronal recordings.

In addition to HG enhancement we consistently found HG decreases relative to a pre-trial baseline. One sensor cluster was unilateral during observation and bilateral during execution, covered ventral sensorimotor cortices, and presumably captured HG activity from ventral higher-tier motor planning areas. During the retention interval we found this relative reduction of HG activity in additional sensors over parietal areas. However, it is not so clear whether these lateral and parietal HG-reductions reflect similar functions. The lateral HG reduction relative to baseline was present in all three phases, observation, retention and execution with some variation in the strength of the effect. This suggests that it is related to the motor task rather than any of the three phases of the task. This interpretation is further corroborated by the observation that it first occurs when the specific grasp was introduced. The effect could be accounted for by the results of monkey single cells studies recording from mirror neurons in ventral premotor areas F5 and F6 [54, 55]. Gallese et al. [54] report that a substantial proportion of the mirror neurons exhibited strict tuning to specific actions. They fired only for their preferred grasp and had reduced spontaneous activity for non-preferred actions. Qualitatively similar effects were reported by Kraskov et al. [55] for mirror neurons with pyramidal tract projections. Accordingly, the reduction of HG activity we found in lateral MEG sensors could result from a net reduction over the population HG activity of neurons with high selectivity for specific actions.

The parietal HG-decrease, however, exhibits different characteristics than the lateral. There, the HG activity is increased relative to baseline during action observation and execution but decreased during the retention interval below pre-trial baseline. Similarly, Perry et al. [7] reported HG-activity increases in the *pure mirroring* electrodes only during observation and execution phases while HG activity decreased during retention. These *pure mirroring* electrodes were more abundant over parietal cortices in their study. Moreover, activation increases in parietal cortex during reach-to-grasp movements has been reported in previous PET and fMRI studies [17, 56], in particular in imitation paradigms. This activation has been interpreted in terms of matching an observed action onto an internal motor representation [17, 57]. Conversely, the HG activation reduction during the retention interval may reflect a mechanism for withholding the execution of the reach-to-grasp movement during the delay [21, 55] as parietal areas play a role in the coordination of vision and motor related brain areas for reach-to-grasp movements [58, 59].

Perry et al. [7], recording ECoG using a similar experimental paradigm, denoted electrodes *mnenomic mirroring* electrodes when the HG activation level was elevated during all three phases, observation, retention, and grasping. We did not find sensors with elevated HG activity in all three phases. However, we did find consistently decreased HG activity over all three intervals in sensors over inferior frontal and parietal areas. Since these sensors show the same response direction during all three phases, they could be termed *mnemonic mirror sites*. In MEG HG-decreases have been reported in working memory tasks starting with the encoding phase [60]. However, it is unclear why the MEG should measure HG decreases when ECoG measurements report increases. One reason could be the much lower spatial resolution of the MEG which is in the order of centimeters while ECoG can resolve functional variations in the order of a few millimeters [61]. In the study of Perry et al. [7] *mnenomic mirror* electrodes where spatially interspersed with *pure mirroring* electrodes and MEG is not capable of resolving such fine structure in functional organization.

Accurate localization of task-related modulations in the HG range during movement observation and execution is important to facilitate further research on functional aspects of the human mirror system. We used sensor sensitivity maps that were calculated on the individual anatomy and then morphed to a standard brain for visualization of the potential brain sources. The analyses presented here are based on planar gradiometers which are most sensitive to brain activation directly under the sensors. However, due to the underlying physics and individual cortical folding patterns, a gradiometers sensitivity profile has a spatial spread on the cortical sheet which depends on the orientation and distance of the cortical area. The sensitivity maps provided here can be interpreted as probability maps for the anatomical location of the sources of the effects observed in the gradiometer sensors. An advantage of this approach is that the sensitivity maps are only based on a biophysical model of how the sensor signal is generated. They do not require complex fitting of inverse models.

Another benefit of our study is the use of natural grasping movements with ecological validity. We used objects of everyday use (bottle, cup, and pen) combined with the grasp types that would naturally be used to pick up and use these objects: whole-hand power-grip for the bottle, handle grip for the cup and precision pinch grip with index finger and thumb for the pen. The spatial patterns we found could guide brain-computer interfaces (BCIs) based on HG activity in the future. It has been argued that areas coding specific goal-directed actions might be well suited for BCIs, for example in posterior parietal cortex [62] which we also found to be modulated by our task. In addition, a better spatial understanding of the human mirror neuron system could benefit advanced neurorehabilitation approaches that show promising results of action observation treatment in areas with mirror properties [63, 64] with potentially greater benefits when combined with motor imagery [65].

Moreover, we were able to include the self-paced nature of the movements into our analysis, not only by looking at the actual start of the movement, but also by analyzing specific time-points during the reach-and-grasp movement using our custom made MEG-compatible movement tracking system, especially the time point of the actual grasp. This enabled us to separate segments into a pre-grasp period, where we expect the pre-shaping of the hand and the grasp to happen [46], and a post-grasp period, where the object is simply held with minimal additional movement of the arm and hand. Using this separation, we were are able to differentiate between grasp placement activity and activity during holding the grasp. From a neuronal perspective there are, at least in monkeys, “grasping neurons” which would respond during the pre-grasp period and stop firing when the grasp is placed around the object and “holding neurons” which only respond when an object us kept in the hand [54]. Such neurons could partly drive the difference in activity patterns we found when comparing pre-grasp and post-grasp periods.

In conclusion, our MEG results reveal complex HG activation patterns during movement observation and execution which overlap in several cortical areas. We found lateral reduction of HG activity during observation, retention and execution of imitated reach-to-grasp movements to natural objects. This activation showed features of *mnenomic mirror* activation. Moreover we found mirror-like HG enhancements during observation and execution in sensors over parietal and sensorimotor areas. In parietal areas this activation decreased below baseline level during retention, supporting the notion of a role of parietal areas in controlling the execution of a planned movement. These results provide further insights into human mirror system localization and response properties in the HG range.

## References

[1] di Pellegrino G, Fadiga L, Fogassi L, Gallese V, Rizzolatti G. Understanding motor events: a neurophysiological study. Exp Brain Res. 1992;91(1):176–80. doi: 10.1007/BF00230027 1301372

[2] Rizzolatti G, Sinigaglia C. The functional role of the parieto-frontal mirror circuit: Interpretations and misinterpretations. Nat Rev Neurosci. 2010;11(4):264–74. doi: 10.1038/nrn2805 20216547

[3] Rizzolatti G, Fogassi L, Gallese V. Neurophysiological mechanisms underlying the understanding and imitation of action. Nat Rev Neurosci. 2001 Sep;2(9):661–70. doi: 10.1038/35090060 11533734

[4] Mukamel R, Ekstrom AD, Kaplan J, Iacoboni M, Fried I. Single-Neuron Responses in Humans during Execution and Observation of Actions. Curr Biol. 2010 Apr 27;20(8):750–6. Available from: doi: 10.1016/j.cub.2010.02.045 20381353 PMC2904852

[5] Pineda JA. The functional significance of mu rhythms: Translating “seeing” and “hearing” into “doing.” Brain Res Rev. 2005;50(1):57–68. doi: 10.1016/j.brainresrev.2005.04.005 15925412

[6] Kilner JM, Marchant JL, Frith CD. Relationship between activity in human primary motor cortex during action observation and the mirror neuron system. PLoS One. 2009;4(3). doi: 10.1371/journal.pone.0004925 19290052 PMC2654140

[7] Perry A, Stiso J, Chang EF, Lin JJ, Parvizi J, Knight RT. Mirroring in the human brain: Deciphering the spatial-Temporal patterns of the human mirror neuron system. Cereb Cortex. 2018;28(3):1039–48. doi: 10.1093/cercor/bhx013 28137724 PMC6059139

[8] Babiloni C, Del Percio C, Vecchio F, Sebastiano F, Di Gennaro G, Quarato PP, et al. Alpha, beta and gamma electrocorticographic rhythms in somatosensory, motor, premotor and prefrontal cortical areas differ in movement execution and observation in humans. Clin Neurophysiol. 2016 Jan;127(1):641–54. doi: 10.1016/j.clinph.2015.04.068 26038115

[9] Buccino G, Vogt S, Ritzl A, Fink GR, Zilles K, Freund HJ, et al. Neural circuits underlying imitation learning of hand actions: An event-related fMRI study. Neuron. 2004 Apr;42(2):323–34. doi: 10.1016/s0896-6273(04)00181-3 15091346

[10] Vogt S, Buccino G, Wohlschläger AM, Canessa N, Shah NJ, Zilles K, et al. Prefrontal involvement in imitation learning of hand actions: Effects of practice and expertise. Neuroimage. 2007;37(4):1371–83. doi: 10.1016/j.neuroimage.2007.07.005 17698372

[11] Castiello U, Begliomini C. The cortical control of visually guided grasping. Neuroscientist. 2008;14(2):157–70. doi: 10.1177/1073858407312080 18219055

[12] Vesia M, Crawford JD. Specialization of reach function in human posterior parietal cortex. Exp Brain Res. 2012;221(1):1–18. doi: 10.1007/s00221-012-3158-9 22777102

[13] Cavina-Pratesi C, Monaco S, Fattori P, Galletti C, McAdam TD, Quinlan DJ, et al. Functional magnetic resonance imaging reveals the neural substrates of arm transport and grip formation in reach-to-grasp actions in humans. J Neurosci. 2010;30(31):10306–23. doi: 10.1523/JNEUROSCI.2023-10.2010 20685975 PMC6634677

[14] Breveglieri R, de Vitis M, Bosco A, Galletti C, Fattori P. Interplay between grip and vision in the monkey medial parietal lobe. Cereb Cortex. 2018 Jun 1 [cited 2021 Jul 12];28(6):2028–42. doi: 10.1093/cercor/bhx109 28472262

[15] Breveglieri R, Bosco A, Galletti C, Passarelli L, Fattori P. Neural activity in the medial parietal area V6A while grasping with or without visual feedback. Sci Rep. 2016;6(1):28893. doi: 10.1038/srep28893 27381869 PMC4933874

[16] Fattori P, Breveglieri R, Bosco A, Gamberini M, Galletti C. Vision for prehension in the medial parietal cortex. Cereb Cortex. 2017;27(2):1149–63. doi: 10.1093/cercor/bhv302 26656999

[17] Iacoboni M, Woods RP, Brass M, Bekkering H, Mazziotta JC, Rizzolatti G. Cortical mechanisms of human imitation. Science. 1999;286(5449):2526–8. doi: 10.1126/science.286.5449.2526 10617472

[18] Caspers S, Zilles K, Laird AR, Eickhoff SB. ALE meta-analysis of action observation and imitation in the human brain. Neuroimage. 2010;50(3):1148–67. doi: 10.1016/j.neuroimage.2009.12.112 20056149 PMC4981639

[19] Leszczyński M, Barczak A, Kajikawa Y, Ulbert I, Falchier AY, Tal I, et al. Dissociation of broadband high-frequency activity and neuronal firing in the neocortex. Sci Adv. 2020 Aug;6(33):eabb0977. doi: 10.1126/sciadv.abb0977 32851172 PMC7423365

[20] Gazzola V, Keysers C. The observation and execution of actions share motor and somatosensory voxels in all tested subjects: Single-subject analyses of unsmoothed fMRI data. Cereb Cortex. 2009;19(6):1239–55. doi: 10.1093/cercor/bhn181 19020203 PMC2677653

[21] Cross KA, Iacoboni M. Neural systems for preparatory control of imitation. Philos Trans R Soc B Biol Sci. 2014;369(1644). doi: 10.1098/rstb.2013.0176 24778373 PMC4006179

[22] Lachaux J-P, Axmacher N, Mormann F, Halgren E, Crone NE. High-frequency neural activity and human cognition: past, present and possible future of intracranial EEG research. Prog Neurobiol [Internet]. 2012;98(3):279–301. doi: 10.1016/j.pneurobio.2012.06.008 22750156 PMC3980670

[23] Manning JR, Jacobs J, Fried I, Kahana MJ. Broadband shifts in local field potential power spectra are correlated with single-neuron spiking in humans. J Neurosci. 2009 Oct;29(43):13613–20. doi: 10.1523/JNEUROSCI.2041-09.2009 19864573 PMC3001247

[24] Ray S, Maunsell JHR. Different origins of gamma rhythm and high-gamma activity in macaque visual cortex. PLoS Biol. 2011;9(4). doi: 10.1371/journal.pbio.1000610 21532743 PMC3075230

[25] Huo X, Wang Y, Kotecha R, Kirtman EG, Fujiwara H, Hemasilpin N, et al. High gamma oscillations of sensorimotor cortex during unilateral movement in the developing brain: A MEG study. Brain Topogr. 2011;23(4):375–84. doi: 10.1007/s10548-010-0151-0 20577795

[26] Cheyne D, Bells S, Ferrari P, Gaetz W, Bostan AC. Self-paced movements induce high-frequency gamma oscillations in primary motor cortex. Neuroimage. 2008;42(1):332–42. doi: 10.1016/j.neuroimage.2008.04.178 18511304

[27] Hinkley LBN, Nagarajan SS, Dalal SS, Guggisberg AG, Disbrow EA. Cortical temporal dynamics of visually guided behavior. Cereb Cortex. 2011;21(3):519–29. doi: 10.1093/cercor/bhq102 20601397 PMC3041007

[28] Muthukumaraswamy SD. High-frequency brain activity and muscle artifacts in MEG/EEG: A review and recommendations. Front Hum Neurosci. 2013;7(MAR):138. doi: 10.3389/fnhum.2013.00138 23596409 PMC3625857

[29] Oldfield RC. The assessment and analysis of handedness: The Edinburgh inventory. Neuropsychologia. 1971;9(1):97–113. doi: 10.1016/0028-3932(71)90067-4 5146491

[30] Shirinbayan SI, Rieger JW. An MR-compatible gyroscope-based arm movement tracking system. J Neurosci Methods. 2017;280:16–26. doi: 10.1016/j.jneumeth.2017.01.015 28147250

[31] Shirinbayan SI, Dreyer AM, Rieger JW. Cortical and subcortical areas involved in the regulation of reach movement speed in the human brain: An fMRI study. Hum Brain Mapp. 2019;40(1):151–62. doi: 10.1002/hbm.24361 30251771 PMC6865438

[32] Dale AM, Fischl B, Sereno MI. Cortical surface-based analysis: I. Segmentation and surface reconstruction. Neuroimage [Internet]. 1999;9(2):179–94. doi: 10.1006/nimg.1998.0395 9931268

[33] Bauer M, Oostenveld R, Peeters M, Fries P. Tactile spatial attention enhances gamma-band activity in somatosensory cortex and reduces low-frequency activity in parieto-occipital areas. J Neurosci. 2006;26(2):490–501. doi: 10.1523/JNEUROSCI.5228-04.2006 16407546 PMC6674422

[34] Muthukumaraswamy SD. Functional properties of human primary motor cortex gamma oscillations. J Neurophysiol. 2010;104(5):2873–85. doi: 10.1152/jn.00607.2010 20884762

[35] Dalal SS, Guggisberg AG, Edwards E, Sekihara K, Findlay AM, Canolty RT, et al. Five-dimensional neuroimaging: Localization of the time-frequency dynamics of cortical activity. Neuroimage. 2008;40(4):1686–700. doi: 10.1016/j.neuroimage.2008.01.023 18356081 PMC2426929

[36] Miller KJ, Honey CJ, Hermes D, Rao RPN, denNijs M, Ojemann JG. Broadband changes in the cortical surface potential track activation of functionally diverse neuronal populations. Neuroimage. 2014;85:711–20. doi: 10.1016/j.neuroimage.2013.08.070 24018305 PMC4347924

[37] Benjamini Y, Hochberg Y. Controlling the False Discovery Rate: A Practical and Powerful Approach to Multiple Testing. J R Stat Soc Ser B. 1995;57(1):289–300.

[38] Gramfort A, Luessi M, Larson E, Engemann DA, Strohmeier D, Brodbeck C, et al. MEG and EEG data analysis with MNE-Python. Front Neurosci. 2013;7(7 DEC):267. doi: 10.3389/fnins.2013.00267 24431986 PMC3872725

[39] Malmivuo J, Plonsey R. Bioelectromagnetism: Principles and Applications of Bioelectric and Biomagnetic Fields. New York: Oxford University Press; 1995. doi: 10.3109/03091909509030281

[40] Yousry T a, Schmid UD, Alkadhi H, Schmidt D, Peraud a, Buettner a, et al. Localization of the motor hand area to a knob on the precentral gyrus. A new landmark. Brain. 1997;120 (Pt 1:141–57. doi: 10.1093/brain/120.1.141 9055804

[41] Cheyne D, Weinberg H. Neuromagnetic fields accompanying unilateral finger movements: pre-movement and movement-evoked fields. Exp Brain Res. 1989;78(3):604–12. doi: 10.1007/BF00230248 2612603

[42] Pfurtscheller G, Lopes Da Silva FH. Event-related EEG/MEG synchronization and desynchronization: Basic principles. Clin Neurophysiol. 1999;110(11):1842–57. doi: 10.1016/s1388-2457(99)00141-8 10576479

[43] Cheyne D. MEG studies of sensorimotor rhythms: A review. Exp Neurol. 2013;245:27–39. doi: 10.1016/j.expneurol.2012.08.030 22981841

[44] Darvas F, Rao RPN, Murias M. Localized high gamma motor oscillations respond to perceived biologic motion. J Clin Neurophysiol. 2013;30(3):299–307. doi: 10.1097/WNP.0b013e3182872f40 23733096 PMC3675661

[45] Crone NE, Miglioretti DL, Gordon B, Lesser RP. Functional mapping of human sensorimotor cortex with electrocorticographic spectral analysis. II. Event-related synchronization in the gamma band. Brain. 1998 Dec;121(12):2301–15. doi: 10.1093/brain/121.12.2301 9874481

[46] Jeannerod M. Intersegmental coordination during reaching at natural visual objects. In: Long JB, Baddeley AD, editors. Attention and Performance IX. Hillsdale, NJ: Lawrence Erlbaum; 1981. p. 153–68.

[47] Cattaneo L, Rizzolatti G. The mirror neuron system. Arch Neurol. 2009;66(5):557–60. doi: 10.1001/archneurol.2009.41 19433654

[48] Breveglieri R, Vaccari FE, Bosco A, Gamberini M, Fattori P, Galletti C. Neurons Modulated by Action Execution and Observation in the Macaque Medial Parietal Cortex. Curr Biol. 2019;29(7):1218–1225.e3. doi: 10.1016/j.cub.2019.02.027 30880012

[49] Dum RP, Strick PL. Motor areas in the frontal lobe of the primate. Physiol Behav. 2002;77(4–5):677–82. doi: 10.1016/s0031-9384(02)00929-0 12527018

[50] Takahashi K, Best MD, Huh N, Brown KA, Tobaa AA, Hatsopoulos NG. Encoding of both reaching and grasping kinematics in dorsal and ventral premotor cortices. J Neurosci. 2017;37(7):1733–46. doi: 10.1523/JNEUROSCI.1537-16.2016 28077725 PMC5320606

[51] Jeannerod M. Visuomotor channels: Their integration in goal-directed prehension. Hum Mov Sci. 1999;18(2–3):201–18.

[52] Begliomini C, De Sanctis T, Marangon M, Tarantino V, Sartori L, Miotto D, et al. An investigation of the neural circuits underlying reaching and reach-to-grasp movements: From planning to execution. Front Hum Neurosci. 2014;8(SEP):1–14. 25228872 10.3389/fnhum.2014.00676 PMC4151344

[53] Karl JM, Whishaw IQ. Different evolutionary origins for the Reach and the Grasp: An explanation for dual visuomotor channels in primate parietofrontal cortex. Front Neurol. 2013;4(208):1–13. 24391626 10.3389/fneur.2013.00208 PMC3870330

[54] Gallese V, Fadiga L, Fogassi L, Rizzolatti G. Action recognition in the premotor cortex. Brain. 1996;119(2):593–609. 8800951 10.1093/brain/119.2.593

[55] Kraskov A, Dancause N, Quallo MM, Shepherd S, Lemon RN. Corticospinal Neurons in Macaque Ventral Premotor Cortex with Mirror Properties: A Potential Mechanism for Action Suppression? Neuron. 2009;64(6):922–30. doi: 10.1016/j.neuron.2009.12.010 20064397 PMC2862290

[56] Grèzes J, Costes N, Decety J. Top-down effect of strategy on the perception of human biological motion: A pet investigation. Cogn Neuropsychol. 1998;15(6–8):553–82. doi: 10.1080/026432998381023 22448838

[57] Rizzolatti G, Craighero L. The mirror-neuron system. Annu Rev Neurosci. 2004;27:169–92. doi: 10.1146/annurev.neuro.27.070203.144230 15217330

[58] Ferrari PF, Bonini L, Fogassi L. From monkey mirror neurons to primate behaviours: Possible “direct” and “indirect” pathways. Philos Trans R Soc B Biol Sci. 2009;364(1528):2311–23. doi: 10.1098/rstb.2009.0062 19620103 PMC2865083

[59] Davare M, Kraskov A, Rothwell JC, Lemon RN. Interactions between areas of the cortical grasping network. Curr Opin Neurobiol. 2011;21(4):565–70. doi: 10.1016/j.conb.2011.05.021 21696944 PMC3437559

[60] Brookes MJ, Wood JR, Stevenson CM, Zumer JM, White TP, Liddle PF, et al. Changes in brain network activity during working memory tasks: A magnetoencephalography study. Neuroimage. 2011;55(4):1804–15. doi: 10.1016/j.neuroimage.2010.10.074 21044687 PMC6485426

[61] Chang EF, Rieger JW, Johnson K, Berger MS, Barbaro NM, Knight RT. Categorical Speech Representation in the Human Superior Temporal Gyrus. Nat Neurosci. 2010;13(11):1428–32. doi: 10.1038/nn.2641 20890293 PMC2967728

[62] Andersen RA, Aflalo T, Kellis S. From thought to action: The brain-machine interface in posterior parietal cortex. Proc Natl Acad Sci U S A. 2019;116(52):26274–9. doi: 10.1073/pnas.1902276116 31871144 PMC6936686

[63] Buccino G. Action observation treatment: A novel tool in neurorehabilitation. Philos Trans R Soc B Biol Sci. 2014;369(1644). doi: 10.1098/rstb.2013.0185 24778380 PMC4006186

[64] Marshall B, Wright DJ, Holmes PS, Williams J, Wood G. Combined action observation and motor imagery facilitates visuomotor adaptation in children with developmental coordination disorder. Res Dev Disabil. 2020;98:103570. doi: 10.1016/j.ridd.2019.103570 31918039

[65] Eaves DL, Riach M, Holmes PS, Wright DJ. Motor imagery during action observation: A brief review of evidence, theory and future research opportunities. Front Neurosci. 2016;10(NOV):514. doi: 10.3389/fnins.2016.00514 27917103 PMC5116576

